# Antioxidant and ACE-Inhibitory Activity of Protein Hydrolysates Produced from Atlantic Sea Cucumber (*Cucumaria frondosa*)

**DOI:** 10.3390/molecules28135263

**Published:** 2023-07-07

**Authors:** Tharindu R. L. Senadheera, Abul Hossain, Deepika Dave, Fereidoon Shahidi

**Affiliations:** 1 Department of Biochemistry, Memorial University of Newfoundland, St. John’s, NL A1C 5S7, Canada; trlsenadheer@mun.ca (T.R.L.S.); abulh@mun.ca (A.H.); 2 Marine Bioprocessing Facility, Centre of Aquaculture and Seafood Development, Fisheries and Marine Institute, Memorial University of Newfoundland, St. John’s, NL A1C 5R3, Canada

**Keywords:** sea cucumber protein hydrolysate, antioxidant activity, ACE inhibition, LDL cholesterol oxidation, DNA scission inhibition

## Abstract

Atlantic sea cucumber is a benthic marine echinoderm found in Northwest Atlantic waters and is harvested mainly for its body wall. The body wall, along with internal organs and aquaphyrangeal bulb/flower, is a rich source of proteins, where the latter parts are often considered as processing discards. The objective of this research was to produce protein hydrolysates from sea cucumber tissues (body wall, flower, and internal organs) with bioactive properties associated with antioxidants, DNA and LDL cholesterol oxidation inhibition, and angiotensin-I-converting enzyme (ACE) inhibitory effects. The protein hydrolysates were prepared using food-grade commercial enzymes, namely Alcalase, Corolase, and Flavourzyme, individually and in combination, and found that the combination of enzymes exhibited stronger antioxidant potential than the individual enzymes, as well as their untreated counterparts. Similar trends were also observed for the DNA and LDL cholesterol oxidation inhibition and ACE-inhibitory properties of sea cucumber protein hydrolysates, mainly those that were prepared from the flower. Thus, the findings of this study revealed potential applications of sea cucumber-derived protein hydrolysates in functional foods, nutraceuticals, and dietary supplements, as well as natural therapeutics.

## 1. Introduction

Sea cucumbers are marine invertebrates that have been consumed as a tonic food and traditional medicine in Asian cultures for centuries. North Atlantic sea cucumber (*Cucumaria frondosa*) has been harvested in recent years for commercial purposes and has gained attention due to its impressive nutritional profile [1]. These marine animals are well-known for their broad range of bioactive compounds, including proteins and protein-derived compounds/products (e.g., protein hydrolysate, peptides, and collagen), lipids (polyunsaturated fatty acids), sulfated glycosaminoglycan (fucosylated chondroitin sulfate and fucoidan), phenolics (phenolic acids and flavonoids), saponins (frondoside A), and carotenoids (astaxanthin and canthaxanthin), which demonstrate a series of biological and pharmacological properties [1,2]. Among them, the phenolics, carotenoids, proteins, sulfated polysaccharides, and saponins of sea cucumbers have been reported to possess strong antioxidant activities [3]. In particular, sea cucumbers have attracted much attention within the scientific community due to their low-fat level and high protein content, rich in essential amino acids (~50%) such as phenylalanine, threonine, isoleucine, and arginine [4].

Nowadays, protein hydrolysates derived from seafood have received tremendous interest because of their unique biological activities and therapeutic potential in clinical treatments. Protein hydrolysates are basically a complex mixture of peptides of various sizes, together with free amino acids, which can be prepared using chemical or enzymatic methods. The production of protein hydrolysates using enzymatic methods is an efficient way to recover bioactive peptides, which exhibit the potential for disease risk reduction and health promotion [2]. Enzymatic hydrolysis could improve functional properties, such as water/oil-holding capacity, solubility, and emulsifying and foaming properties, through influencing the hydrophobicity, molecular size, and polar groups of peptides [5]. Apart from functional properties, protein hydrolysates are involved in numerous biological functions, including antihypertension, antithrombotic, immunomodulatory, anticancer, antioxidant, and antimicrobial activities [6]; for example, protein hydrolysates derived from sea cucumber have shown antioxidant, angiotensin-converting enzyme (ACE) inhibitory, immunomodulatory, and anti-inflammatory properties, among others, which are closely related to the structural properties of hydrolysates/peptides [7,8,9,10,11]. However, the proper choice of enzymes, mainly proteases, plays a key role in whether they exhibit functional properties, as the kind of proteases used can directly affect the characteristics of the final products [10]. For instance, microbial proteases, namely Flavourzyme, Alcalase, and Corolase are very common in industrial applications because of their function, cost, and favorable operational conditions [8].

Reactive oxygen species (ROSs), such as superoxides, peroxides, hydroxyl radicals, and singlet oxygen, cause oxidative stress, mainly when there is an imbalance between the production of free radicals/ROSs and the scavenging capacity of endogenous antioxidants. Excessive generation of ROSs may oxidize proteins, enzymes, DNA, low-density lipoprotein (LDL), lipids, carbohydrates, and membranes, leading to the development of chronic disease conditions. In particular, oxidative stress plays a key role in several chronic ailments, including arthritis, cardiovascular diseases, cancer, diabetes, and neurodegenerative disorders [3,12]. For instance, the oxidation of LDL can cause coronary heart disease, while DNA oxidation plays a key role in carcinogenesis [12]. However, protein hydrolysates/peptides are believed to serve as natural antioxidants that can protect/control oxidative damage and the associated diseases. These are mainly dependent on the degree of hydrolysis, enzyme specificity, and the nature of the peptides, such as amino acid composition, molecular weight, and hydrophobicity [13]. In particular, these can show antioxidant activity by scavenging free radicals, chelating metal ions, inhibiting unsaturated fatty acid autoxidation, or acting as reducing agents. Therefore, this study investigates the antioxidant properties, as well as the DNA oxidation, LDL oxidation, and ACE inhibitory effects, of protein hydrolysates obtained from different body parts and processing discards of Atlantic sea cucumber (*Cucumaria frondosa*) using Alcalase, Corolase, Flavourzyme, and their combination. To the authors’ best knowledge, this is the first study in which the DNA and LDL oxidation inhibitory potential of sea cucumber protein hydrolysates are being investigated. In particular, sea cucumber internal organs, either with or without flower, are processing discards and account for around 50% of sea cucumber biomass. Therefore, it is important to examine the biological activities of the protein hydrolysates obtained from sea cucumber processing discards in order to maximize the utilization of this exotic animal. 

## 2. Results and Discussion

### 2.1. Hydroxyl Radical Scavenging Activity

**Figure 1:**
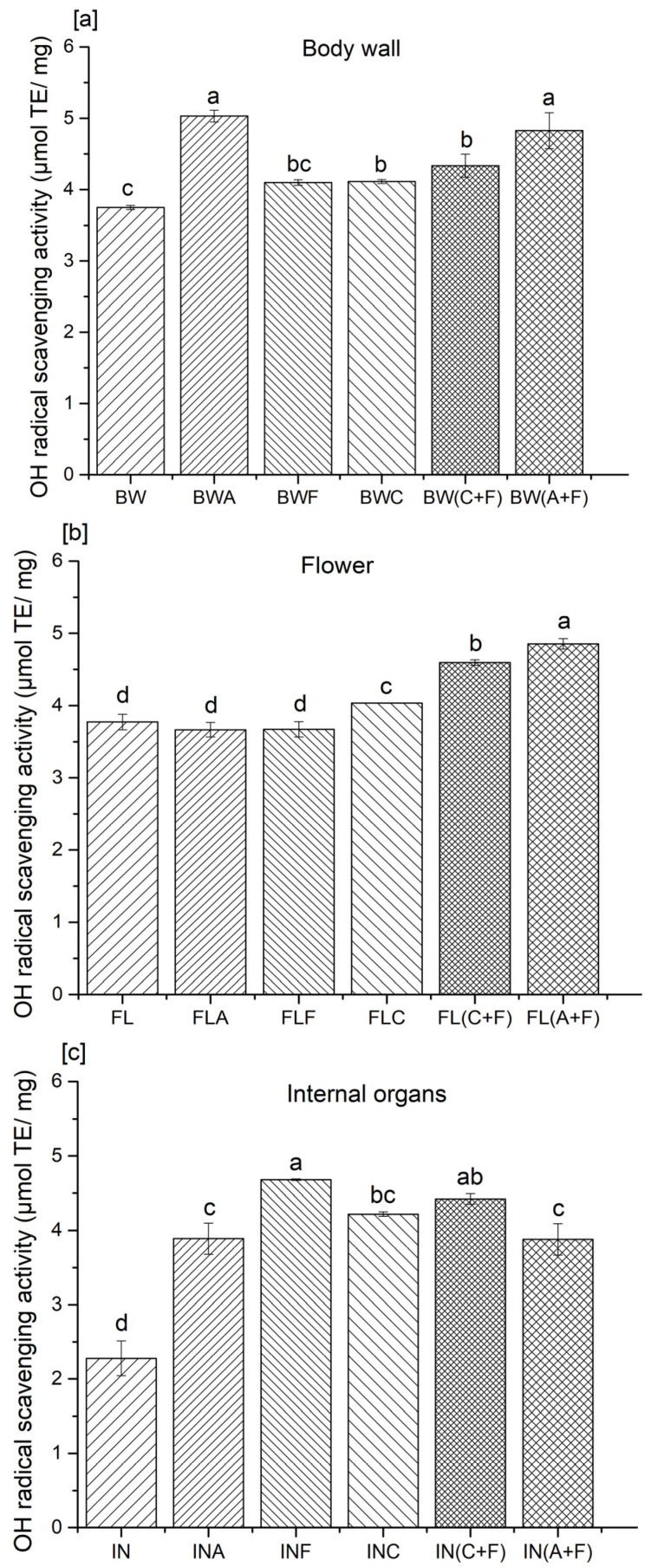
Hydroxyl radical scavenging activity of protein hydrolysates from the (**a**) body wall, (**b**) flower, and (**c**) internal organs of sea cucumber. A—Alcalase; F—Flavourzyme; C—Corolase; A+F—combination of Alcalase and Flavourzyme; C+F—combination of Corolase and Flavourzyme. Different letters on the same concentration of all hydrolysates indicate significant differences at *p* < 0.05.

The hydroxyl radical, an oxygen-derived radical, is the most reactive free radical in biological systems. It can easily react with biomolecules such as proteins (amino acids), DNA, and membrane lipids [14]. Excessive production of hydroxyl radicals may induce cellular damage through oxidative stress. Thus, the removal of excessive levels of hydroxyl radicals is considered as one of the effective defense strategies in preventing the occurrence of numerous cellular disorders, such as cancer, cardiovascular diseases, and diabetes, among others [15]. The scavenging ability of antioxidant substances can be determined using EPR with detection of the spin adduct of 5,5-dimethyl-1-pyrroline N-oxide (DMPO) [16]. In this study, the hydroxyl radical scavenging activity of sea cucumber protein hydrolysates varied between 2.27 and 5.03 µmol TE/mg of protein, and a significant difference (*p* > 0.05) was observed between the enzyme-treated and untreated samples of each sea cucumber body part (Figure 1).

Hydrolysates prepared from the flower, with sequential enzyme treatment with Alcalase and Flavourzyme, showed higher hydroxyl radical scavenging activities compared to all other enzyme treatments in each group. The hydroxyl radical scavenging activity varied between 11 and 27% in terms of percentage. Similar observations were made by Zhang et al. [10], on hydrolysates prepared from Atlantic sea cucumber using Alcalase and trypsin. The authors suggested that enzyme treatments were responsible for the improved antioxidant potential, hydroxyl radical, DPPH radical, and superoxide anion scavenging activities of protein hydrolysates compared to their untreated counterparts. Yan et al. [17] stated that protein hydrolysates obtained from Atlantic sea cucumber scavenge hydroxyl radicals by donating electron/hydrogen and quenching radicals. Ambigaipalan and Shahidi [6] prepared protein hydrolysates from shrimp shell processing discards and found that the hydroxyl radical scavenging activity ranged between 2.54 and 3.9 μmol of carnosine/mg sample. In addition, Girgih et al. [18] developed protein hydrolysates from cod fish and suggested that the presence of hydrophobic amino acids inversely correlates with hydroxyl radical activity. The authors reported that unfractionated protein hydrolysates exhibit higher hydroxyl radical scavenging activity than fractionated peptides obtained from cod protein hydrolysates. These results indicated that the fractionation of peptides might lead to the loss of their synergistic effect toward neutralizing free radicals. In contrast, peptide fractions prepared from hempseed proteins showed higher hydroxyl radical scavenging activity than their corresponding unfractionated hydrolysates [13]. Nonetheless, Cumby et al. [19] stated that the radical scavenging activity of peptides or protein hydrolysates correlated with the hydrogen donor activity of the hydroxyl groups of aromatic amino acid residues (tyrosine, histidine, tryptophan, and phenylalanine). Our previous study found that these amino acids were abundant in the Atlantic sea cucumber’s body wall, flower, and internal organs [8]. The radical scavenging activity of these aromatic amino acid residues improves through resonance stabilization. Hence, the presence and absence of such amino acids in peptides, as well as their positioning in the peptide sequence, also influence the antioxidant activity [6]. For instance, the proper positioning of glutamine, leucine, and histidine can improve the radical scavenging activities in antioxidative peptide sequences [20].

### 2.2. Reducing Power

**Figure 2:**
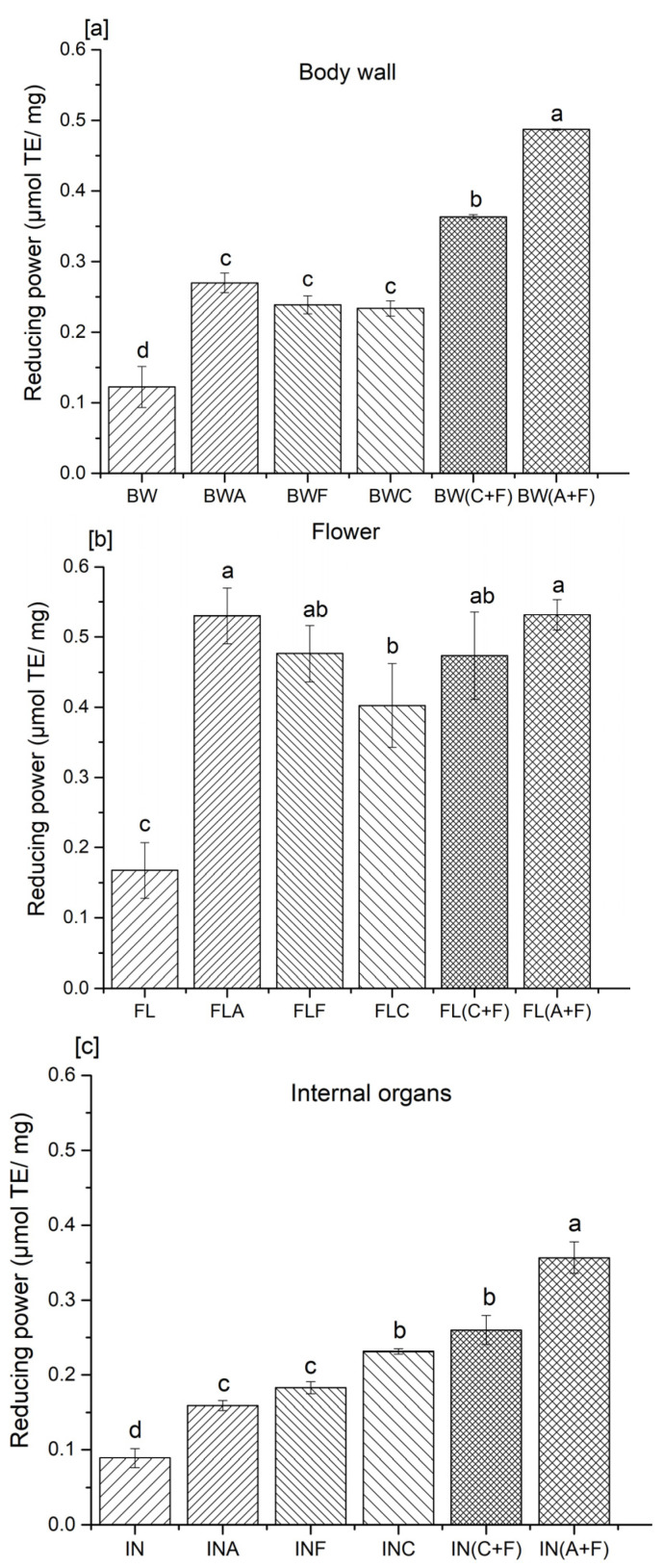
Reducing power of protein hydrolysates from the (**a**) body wall, (**b**) flower, and (**c**) internal organs of sea cucumber. A—Alcalase; F—Flavourzyme; C—Corolase; A+F—combination of Alcalase and Flavourzyme; and C+F—combination of Corolase and Flavourzyme. Different letters on the same concentration of all hydrolysates indicate significant differences at *p* < 0.05.

The ferric-reducing antioxidant power assay is often used to determine the ability of natural antioxidants to donate electron or hydrogen atoms [17]. This assay is categorized under the single electron transfer (SET)-based method, which involves reducing higher valency elements to their lower valence state. In particular, the reduction of the ferric ion (Fe^3+^)–ligand complex to the ferrous (Fe^2+^) complex is monitored with the absorbance at 700 nm [21]. Previous studies have demonstrated a direct correlation between the reducing power and antioxidant activity of protein hydrolysates [13]. Most non-enzymatic antioxidant activities are mediated through redox reactions, including reducing power. Antioxidative peptides in protein hydrolysates have the ability to reduce the Fe^3+^/ferric cyanide complex to the ferrous form. In this study, the reducing power was varied, from 0.12 to 0.48, 0.08 to 0.35, and 0.16 to 0.53 μmol of Trolox/mg of proteins in the body wall, internal organs, and flower hydrolysates, respectively (Figure 2).

Samples treated with a combination of Alcalase and Flavourzyme showed significantly higher (*p* < 0.05) reducing power in all three analyzed body parts of sea cucumber (0.48, 0.53, and 0.35 μmol of Trolox/mg protein for the body wall, flower, and internal organs, respectively) compared to their other-treated and untreated counterparts. The trend for sea cucumber samples hydrolyzed with the combination of Alcalase and Flavourzyme on reducing power was similar to that observed for the radical scavenging activity of tested samples. The current results are supported by the findings of Wiriyaphan et al. [22] and Chalamaiah et al. [23], who indicated that ferric-reducing antioxidant power was directly influenced by the type of protease used to prepare protein hydrolysates from threadfin bream surimi byproducts and common carp roe. Yan et al. [17] reported that the reducing power of sea cucumber viscera was also influenced by the specificity of the enzyme employed. The results indicated that samples hydrolyzed with Alcalase, Flavourzyme, and trypsin possessed greater reducing power than those prepared using bromelain, pepsin, and papain. They suggested that the differences in the activity may be attributed to the presence of hydrophobic amino acids or peptides that can react with free radicals to form more stable products. Cumby et al. [19] further explained this by suggesting that the reducing power and other radical scavenging abilities of protein hydrolysates are composition dependent, and may vary depending on the protease employed in the hydrolysis process. The strong reducing power of protein hydrolysates is associated with the increased availability of hydrogen atoms and electrons due to the liberation of the peptides during the hydrolysis process [23]. These peptides could prevent the propagation of radical chain reactions. According to Udenigwe et al. [24], electron donation ability in amino acid residues, including the sulfhydryl group of cysteine, also contributes to the reducing capacity of peptides. Therefore, the presence of the sulfhydryl group, or its oxidized forms, has a direct impact on the reducing capacity of protein hydrolysates.

### 2.3. Inhibition of Peroxyl and Hydroxyl Radical-Induced Supercoiled DNA Strand Scission

Irreversible modification of DNA due to oxidative damage may lead to mutation, carcinogenesis, and other pathological processes. Free radicals generated in living cells could mediate the base modification of DNA, production of base-free sites, DNA strand breakage, abnormal chromosomal arrangements, and DNA–protein cross-links, among others [12]. Most reactive free radicals, such as hydroxyl and peroxyl radicals, possess greater reduction potential that can react with biomolecules, including damaging DNA at both the phosphate backbone and the nucleotide bases. For example, hydroxyl radicals can abstract a hydrogen atom from pyrimidine and purine bases, as well as from the deoxyribose sugar moieties of DNA [12,25]. Thus, it is crucial to suppress DNA oxidation in order to avoid lethal damage to living cells. The current assay determines the inhibitory activity of antioxidants against hydroxyl and peroxyl radical-induced DNA strand scission in supercoiled plasmid pBR3222. The supercoiled DNA (form I) could alter its conformation to a nicked open circular form (form II) or a linear form (form III) as a consequence of the oxidation of DNA induced by free radicals [6]. The inhibitory activity of antioxidants against DNA strand scission is evaluated by considering the levels of intact DNA strand and nicked DNA fractions, using agarose gel electrophoresis [21]. In general, the linear form of DNA showed restricted movements through the agarose gel network compared to the supercoiled DNA.

**Figure 3:**
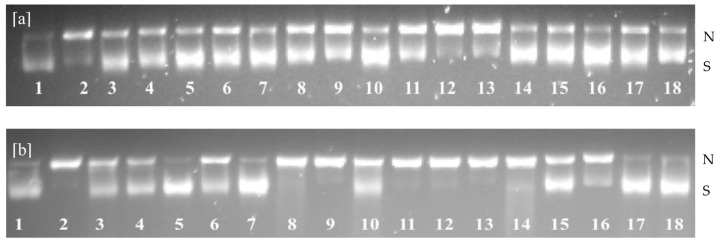
Agarose gel electrophoresis of the inhibition of hydroxyl radical- (**a**) and peroxyl radical-s (**b**) induced DNA scission by hydrolysates from the body wall (BW), internal organs (IN), and flower (FL) of sea cucumber. N—Nicked DNA; S—Supercoiled DNA; 1—Blank; 2—Control; 3—BWA; 4—BWF; 5—BWC; 6—BW(C+F); 7—BW(A+F); 8—INA; 9—INF; 10—INC; 11—IN(C+F); 12—IN(A+F); 13—FLA; 14—FLF; 15—FLC; 16—FL(C+F); 17—FL(A+F); and 18—Carnosine.

Sea cucumber protein hydrolysates were assessed for their protective effect against hydroxyl and peroxyl radical-induced strand scission. Carnosine, a β-alanyl-histidine dipeptide, is used as a positive control due to its physiological relevance as a natural antioxidant in muscle proteins. It is present in skeletal muscles at millimolar concentrations [26]. In these experiments, no clear trends were observed between single-enzyme-treated (exopeptidase or endopeptidase) and combined-enzyme-treated sea cucumber protein hydrolysates in inhibiting both hydroxyl and peroxyl radical-mediated DNA strand scission. However, body wall and flower treated with Alcalase and Flavourzyme showed similar activity when compared to carnosine (Figure 3). The inhibition of DNA scission, induced by hydroxyl radicals, ranged from 29.61 to 77.22% in body wall, 35.77 to 58.49% in internal organ, and 37.64 to 76.94% in flower hydrolysates (Table 1).

Moreover, the inhibitory effect of peroxyl radical-induced DNA oxidation varied from 56.11 to 81.85% in body wall, 32.90 to 61.53% in internal organ, and 57.41 to 79.79% in flower hydrolysates. Similar observations were reported by Ambigaipalan and Shahidi [26] for date seed protein hydrolysates, where the retention levels of supercoiled DNA were 13–33 and 47–83% for hydroxyl and peroxyl radical effects, respectively. These findings were also in accordance with the inhibitory activity of supercoiled DNA oxidation indicated for blacktip shark gelatin hydrolysates [27]. The differences between reported values for hydroxyl and peroxyl radical activities could be due to the half-life of each radical. The hydroxyl radical is an extremely reactive species with a shorter half-life compared to the peroxyl radical, which has a relatively long half-life and a high affinity for diffusion into cells [27]. Thus, the protective effect of sea cucumber protein hydrolysates was possibly due to the chelation of metal ions and scavenging activity of hydrolysates. Moreover, Lassoued et al. [28] stated that DNA strand breakage could be due to the hydrophobic peptides. The inhibitory activity of hydrophobic scavengers in preventing plasmid DNA damage is more effective than that of hydrophilic scavengers. Kittiphattanabawon et al. [27] suggested that the biological activities of protein hydrolysates are determined by several factors, including their constituent amino acids and peptides, as well as their sequence, size, and configuration, among others. This is the first study of DNA oxidation inhibition for any species of sea cucumber protein hydrolysates; thus, a direct relationship with the literature data is not possible.

### 2.4. Inhibition of Cupric Ion-Induced Human Low-Density Lipoprotein (LDL) Peroxidation

Plasma low-density lipoprotein (LDL) oxidation, caused by the action of metal ions or reactive oxygen species, is one of the major risk factors for the development of atherosclerosis, which is the primary cause of a majority of cardiovascular diseases [29]. Therefore, inhibition of LDL oxidation can provide an effective strategy for preventing cardiovascular diseases. The copper-induced LDL oxidation assay determines the oxidative susceptibility of LDL by monitoring the formation of CDs, the initiation phase of LDL oxidation [30]. The increase in LDL oxidation is indicated by a change in the absorbance of CD, at 234 nm. The increase in CDs is linked to the formation of cholesteryl linoleate hydroperoxides and cholesteryl linoleate hydroxides [27]. Thus, the presence of both compounds may create a favorable condition for lipid peroxidation [26].

**Figure 4:**
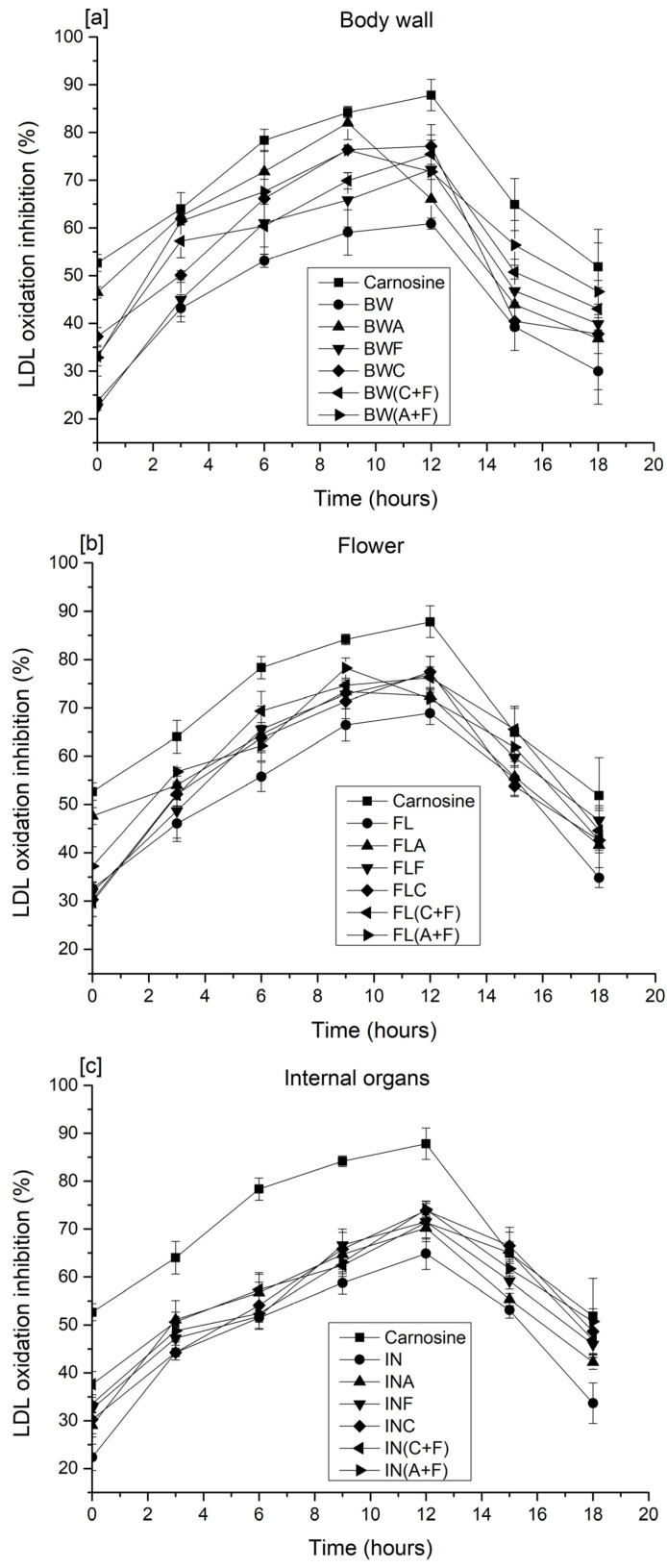
LDL cholesterol oxidation of sea cucumber protein hydrolysates prepared from (**a**) the body wall, (**b**) flower, and (**c**) internal organs. A—Alcalase; F—Flavourzyme; C—Corolase; A+F—combination of Alcalase and Flavourzyme; and C+F—combination of Corolase and Flavourzyme.

Figure 4 shows the inhibition of copper-induced LDL oxidation by carnosine and sea cucumber protein hydrolysates over 18 h of incubation at 37 ºC. Inhibition of LDL cholesterol oxidation of carnosine ranged from ~53 to ~88%. The inhibitory activity of protein hydrolysates prepared from the body wall ranged from ~23 to ~82%, whereas the inhibitory activity of hydrolysates prepared from the flower was ~30 to ~79%. The highest inhibition observed from protein hydrolysates prepared from internal organs was ~74% after 12 h of incubation, when hydrolysates were prepared using Alcalase and Flavourzyme. Carnosine exhibited the highest efficacy in inhibiting cupric ion-mediated LDL oxidation (87%) after 12 h, while the untreated counterparts of sea cucumber samples showed the lowest inhibitory activity in all three groups (BW, FL, and IN). Protein hydrolysates from each group showed strong inhibitory activity against LDL oxidation compared to the untreated samples. This could be due to the chelating ability and free radical scavenging activity of peptides present in the protein hydrolysates [6]. Kittiphattanabawon et al. [27] reported that gelatin hydrolysates prepared from blacktip shark gelatin inhibited human LDL oxidation by 8-39%. Amino acid composition plays a vital role in preventing LDL oxidation. The availability of amino acid residues, including tyrosine, histidine, phenylalanine, methionine, glycine, proline, or leucine, may enhance the chelating and radical scavenging activities of peptides. Park et al. [31] further explained that peptides containing these amino acids render stronger antioxidant activity against lipid peroxidation than do individual amino acids. A recent study about the influence of peptide charge on the inhibition of LDL oxidation revealed that the positively charged peptide fractions possess significantly higher inhibitory activity than the negatively charged fractions do [29]. The effectiveness of amino acid charge on the inhibition of LDL oxidation was observed by Wang et al. [32]. These authors suggested that peptides with negatively charged amino acids could prolong the lag time of LDL oxidation. However, the negatively charged amino acids could also chelate metal ions and may be involved in terminating the free radical chain reaction, as well as in preventing the propagation of LDL oxidation [33]. The highest inhibitory activities were observed in the protein hydrolysates treated with a combination of enzymes (Alcalase + Flavourzyme and Corolase + Flavourzyme). Similar inhibitory activity against LDL oxidation was reported for date seed protein hydrolysates [26]. The authors noted that lipophilic antioxidants might extend the LDL oxidation lag phase induced by metal ions. Nevertheless, the exact mechanism of LDL oxidation inhibition by protein hydrolysates is not yet fully understood. To date, this is the first study performed that evaluates the potential inhibitory activity of LDL oxidation for sea cucumber protein hydrolysates; thus, a direct relationship with other sea cucumber species is not feasible.

### 2.5. Angiotensin I Converting Enzyme (ACE) Inhibitory Activity

**Figure 5:**
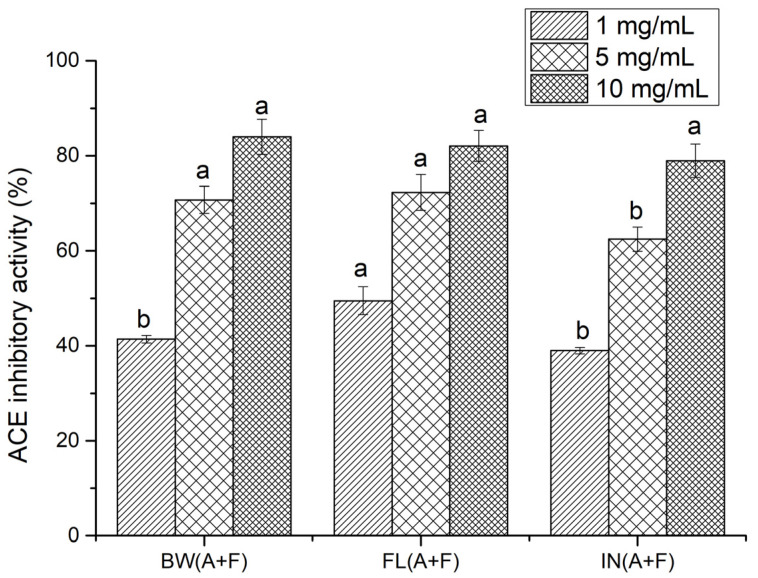
ACE-inhibitory activity (%) of sea cucumber protein hydrolysates (BW—body wall; FL—flower, and IN—internal organs, using A+F—combination of Alcalase and Flavourzyme). Different letters for the same concentration of all hydrolysates indicate significant differences at *p* < 0.05.

ACE-inhibitory activity was determined for the samples that exhibited the highest antioxidant activity following enzyme treatment. Thus, samples digested with a combination of Alcalase and Flavourzyme were selected in order to investigate their antihypertensive activity. Different hydrolysate concentrations, of 10, 5, and 1 mg/mL, were investigated (Figure 5).

No significant difference (*p* > 0.05) was observed in the ACE-inhibitory activities of protein hydrolysates obtained from the body wall, flower, and internal organs at 10 mg/mL. Hydrolysates prepared with the flower exhibited significantly higher (*p* > 0.05) ACE-inhibitory activity than did the body wall and internal organ hydrolysates at 1 mg/mL, while the body wall and flower offered a similar trend at 5 mg/mL. ACE-inhibitory activity was also calculated using IC_50_, and, among all protein hydrolysates, the lowest IC_50_ value (1.06 mg/mL) was observed for flower protein hydrolysates, while internal organ protein hydrolysates showed the highest IC_50_ value, at 1.81 mg/mL. The IC_50_ value for body wall hydrolysates was 1.66 mg/mL. However, the observed IC_50_ values were much higher than that of captopril, a synthetic ACE inhibitor drug, with an IC_50_ of 4.78 × 10^−6^ mg/mL. Compared to synthetic inhibitors, the reported ACE-inhibitory activities of natural peptides are relatively low [34]. However, due to the side effects associated with synthetic drugs, potent natural compounds have received considerable attention for preventing cardiovascular diseases [35]. Our previous study found that sea cucumber (*C. frondosa*) peptides have the potential to inhibit ACE, which was predicted using a range of in silico techniques [2]. Studies on protein hydrolysates from other sea cucumber species, namely *Actinopyga lecanora* [36], *Acaudina molpadioidea* [34], *Stichopus horrens* [37], *Apostichopus japonicas* [11], and *Holothuria atra, Holothuria leucospilota*, and *Bohadschia marmorata* [38], provide some of the most recent information on potent ACE-inhibitory active peptides. The observed IC_50_ values for protein hydrolysates prepared from sea cucumber (*Cucumaria frondosa*) in the present study were lower than those prepared from sea cucumber *S. horrens* [39]. According to Forghani et al. [39], the IC_50_ values were 2.24, 2.28, 2.48, and 6.38 mg/mL for Flavourzyme, trypsin, papain, and Proteomax hydrolysates, respectively, prepared with samples from *S. horrens*. In contrast, Alcalase hydrolysates from the same species showed comparatively lower IC_50_ values (0.48 mg/mL) than the present values. This indicates that the IC_50_ value for the ACE-inhibitory activity of sea cucumber protein hydrolysates may depend on the protease type. Therefore, a comparison of the current data with previous studies is rather difficult, due to discrepancies in hydrolysis conditions, the type of protease used, and other related variables, such as species and geographical location. In addition, most studies were conducted using whole sea cucumber samples, instead of different body parts of the animal. However, the present results are similar to the IC_50_ values obtained from salmon skin collagen hydrolysates (1.165 mg/mL) [40], cuttlefish hydrolysates (1.58 mg/mL) [41], and goby fish protein hydrolysates (1.36–3.33 mg/mL) [42].

Furthermore, it is noteworthy that low-molecular-weight peptide fractions possess greater ACE-inhibitory activity compared to high-molecular-weight peptides and protein hydrolysate mixtures [36,43]. Recent research studies have focused on identifying specific peptides responsible for ACE-inhibitory activity. The reported IC_50_ values are comparatively lower than those of high-molecular-weight peptides and protein hydrolysates [6,34]. This indicates that fractionation strategies, including ultrafiltration, are efficient for enhancing the ACE-inhibitory activities of protein hydrolysates. Potent peptide inhibitors of ACE have distinct structural features, including hydrophobic amino acid residues at the C-terminal and aliphatic amino acid residues at the N-terminal. In general, it was suggested that ACE-inhibitory peptides have short sequences, ranging in length from 2 to 12 amino acids [26,44]. Thus, further studies are needed to identify the specific peptides responsible for the ACE-inhibitory properties of protein hydrolysates prepared from *C. frondosa*.

## 3. Materials and Methods

### 3.1. Preparation of Protein Hydrolysates

Fresh Atlantic sea cucumbers (*Cucumaria frondosa*) were harvested from the northwest and southeast regions of the St. Pierre Bank (NAFO Division 3Ps), Newfoundland, Canada. Alcalase (EC 3.4.21.62, 2.4 AU/g) and Flavourzyme (EC 3.4.11.1, 1000 LAPU/g) were purchased from Novozymes, Bagsvaerd, Denmark. Corolase 7089 (EC 3.4.24.28) was procured from AB enzymes GmbH, Darmstadt, Germany. After harvesting, sea cucumbers were dissected and separated into body wall (BW), internal organs (IN), and flower (FL) samples, followed by freeze-drying. Dried tissues were ground to particle size ≤ 100 μm prior to hydrolysis. Protein hydrolysates were prepared according to the method described previously [8].

### 3.2. Hydroxyl Radical Scavenging Activity

Hydroxyl radical scavenging capacity was determined using an EPR spectrometric (Bruker E-scan, Bruker BioSpin Co., Billerica, MA, USA) method, as described by Hossain et al. [45], with slight modifications. The samples were dissolved in deionized water in order to obtain a final concentration of 10 mg/mL. The sample (200 μL) was mixed with 10 mM H_2_O_2_ (200 μL), 17.6 mM 5,5-dimethyl-1-pyrroline N-oxide (DMPO; 400 μL), and 10 mM FeSO_4_ (200 μL). The mixture was allowed to react for 3 min at room temperature, and then injected into the sample cavity of an electron paramagnetic resonance (EPR) spectrometer (Bruker E-scan, Bruker BioSpin Co., Billerica, MA, USA). Deionized water was used as the control. The EPR spectra were recorded, and Trolox (0–50 μM) was used to prepare the standard curve. The hydroxyl radical scavenging capacity, expressed as micromoles (µM) of Trolox equivalents (TE)/mg of protein hydrolysate, was calculated according to the following equation:

Hydroxyl radical scavenging capacity (%) = (EPR signal intensity for the control − EPR signal intensity for the sample)/EPR signal intensity for the control × 100

### 3.3. Reducing Power

The reducing power of sea cucumber protein hydrolysates was evaluated according to the method described by Cumby et al. [19], with minor modifications. Phosphate buffer (0.2 M, pH 6.6) was used to dissolve sea cucumber protein hydrolysates (0.5 mg/mL). One milliliter of sea cucumber protein hydrolysate was mixed with 2.5 mL of 1% potassium ferricyanide solution, and the mixture was incubated at 50 °C for 20 min. Subsequently, 2.5 mL of 10% trichloroacetic acid (TCA) was added, and the mixture was centrifuged for 10 min at 1000 g. After centrifugation, 2.5 mL of supernatant was mixed with 2.5 mL of deionized water and 0.5 mL of 0.1% ferric chloride solution. The reaction was allowed to proceed for 10 min, and the absorbance of the solution was measured using A UV–visible spectrophotometer (HP 8452 A diode array spectrophotometer, Agilent Technologies, Palo Alto, CA, USA) at 700 nm. The control was prepared without the addition of hydrolysates, while the blank contained only protein hydrolysates. A standard curve was built using varying concentrations (0–1000 µM) of Trolox, and the reducing power was expressed as µM of TE/mg of protein hydrolysate.

### 3.4. Inhibition of Peroxyl and Hydroxyl Radical-Induced Supercoiled DNA Strand Scission

The inhibitory activity of protein hydrolysates against DNA strand scission caused by the action of hydroxyl and peroxyl radicals was determined according to Hossain et al. [25]. Supercoiled plasmid DNA (PBR 322) was dissolved in 0.5 M PBS (pH 7.4), to a final concentration of 50 μg/mL, while protein hydrolysates and carnosine standard were dissolved in distilled water. For the determination of peroxyl radical-induced DNA oxidation, 2 μL of protein hydrolysates (6 mg/mL) were mixed with 4 μL AAPH [2,2′-azobis(2-aminopropane) dihydrochloride, 7 mM], 2 μL of PBS (0.1 M), and 2 μL of DNA (50 μg/mL). In order to produce hydroxyl radicals, 2 μL of FeSO_4_ (0.5 mM) and 2 μL of H_2_O_2_ (0.5 mM) were added to the mixture of protein hydrolysates (2 μL, 0.1 mg/mL), PBS (2 μL, 0.1 M), and DNA (2 μL, 50 μg/mL). The mixture was incubated at 37 °C for 1 h in the dark prior to the addition of 2 μL of loading dye, consisting of 0.25% bromophenol blue, 0.25% xylene cyanol, and 50% glycerol in distilled water. A control (DNA only) and a blank (DNA + free radicals devoid of protein hydrolysates) were prepared for each set of tested samples. Ten microliters of each mixture was then loaded into agarose gel (0.7% *w*/*v*), prepared in Tris-acetic acid-EDTA (TAE) buffer (40 mM Tris-acetate containing 1 mM EDTA, pH 8.5), and stained with SYBR safe. Gel electrophoresis was conducted in a horizontal submarine gel electrophoresis apparatus (Owl Separation Systems Inc., Portsmouth, NH, USA) at 80 V for 90 min. An Alpha-Imager gel documentation system (Cell Biosciences, Santa Clara, CA, USA) was used to visualize the bands under trans-illumination with UV light. The protective effects of protein hydrolysates were determined using the retention percentage of supercoiled DNA strand according to the following equation:

DNA retention (%) = (Area of supercoiled DNA with oxidative radical and protein hydrolysate/Area of supercoiled DNA in control) × 100

### 3.5. Inhibition of Cupric Ion-Induced Human Low-Density Lipoprotein (LDL) Peroxidation

Protein hydrolysates were evaluated for their inhibitory effect of cupric ion-induced human LDL peroxidation, according to the method described by Hossain et al. [30]. Human LDL cholesterol (5 mg/mL) was dialyzed in 10 mM phosphate buffer (PBS; pH 7.4, 0.15 M NaCl) at 4 °C for 18 h. The dialyzed and diluted LDL (0.04 mg/mL) was mixed with protein hydrolysate solutions (0.1 mg/mL) and pre-incubated at 37 °C for 15 min. The oxidation reaction was then initiated by adding 100 μM cupric sulfate, followed by incubation at 37 °C for 12 h. The resultant conjugated dienes (CDs) from human LDL oxidation were measured at 234 nm using a spectrophotometer in 3 h intervals until the end of the incubation period. For each sample, an appropriate blank was prepared, devoid of LDL or CuSO_4_, and carnosine was used as a positive control. The inhibitory effect of the protein hydrolysates on the formation of CDs was calculated using the following equation.

% Inhibition of CD (%) = [(Abs control − Abs sample)/(Abs control − Abs native LDL)] × 100

where Abs control is the absorbance of LDL with CuSO_4_ and PBS; Abs sample is the absorbance of LDL with CuSO_4_ and sample or standard, and Abs native LDL is the absorbance of LDL and PBS only.

### 3.6. Angiotensin I Converting Enzyme (ACE) Inhibitory Activity

Sea cucumber protein hydrolysates exhibiting the highest antioxidant activities from each organ were used to determine ACE-inhibitory activity. The inhibitory activity was determined according to the method of Ambigaipalan and Shahidi [6], with slight modifications. Sodium 4-(2-hydroxyethyl)-1-piperazineethanesulfonate (HEPES)-HCl buffer (50 mM), containing 300 mM NaCl (pH 8.3), was used to dissolve samples and ACE. Twenty-five microliters of ACE solution (0.25 unit/mL) was added to each of sea cucumber protein hydrolysate sample (50 μL, 5 mg/mL), and the mixture was pre-incubated at 37 °C for 5 min. Thereafter, the reaction was initiated by adding 50 μL of hippuryl-L-histidyl-L-leucine (HHL, 6 mg/mL) solution to the mixture, followed by incubation at 37 °C for 15 min. In order to terminate the enzymatic reaction, HCl (125 μL, 1 M) was added, forming hippuric acid, which was extracted with ethyl acetate (1 mL). The mixture was homogenized for 1 min prior to centrifugation at 1200× *g* for 5 min, using an Eppendorf centrifuge (model 5415, Hamburg, Germany). The collected supernatant (1 mL) was placed in boiling water to remove ethyl acetate. The remaining hippuric acid in the tube was dissolved in distilled water (1 mL), and the absorbance was recorded at 228 nm using a spectrophotometer. The control was prepared using HEPES-HCl buffer (50 mM), containing 300 mM NaCl (pH 8.3), devoid of samples. The sample blank and control blank were processed in the same manner, except for that of the ACE solution, which was added into the reaction before the addition of 1 M HCl. The ACE-inhibitory activity (%) was determined according to the following equation.

ACE inhibitory activity (%) =

$$\left\{1\;-\;\frac{\left(\mathrm{Absorbance}\;\mathrm{of}\;\mathrm{sample}\;-\;\mathrm{Absorbance}\;\mathrm{of}\;\mathrm{sample}\;\mathrm{blank}\right)}{\left(\mathrm{Absorbance}\;\mathrm{of}\;\mathrm{control}\;-\;\mathrm{Absorbance}\;\mathrm{of}\;\mathrm{control}\;\mathrm{blank}\right)}\right\}\;\times \;100$$

### 3.7. Statistical Analysis

All of the experiments were carried out in triplicate, and data were reported as mean ± standard deviation. One-way ANOVA was performed, and means were compared with Tukey’s HSD test (*p* < 0.05), using SPSS 16.0 for Windows (SPSS Inc., Chicago, IL, USA).

## 4. Conclusions

This study produced bioactive protein hydrolysates from the body wall and underutilized processing byproducts of Atlantic sea cucumber (*C. frondosa*), using Alcalase (endopeptidase), Flavourzyme (endo- and exopeptidase), and Corolase (endopeptidase), as well as their combination. Samples treated with combinations of Alcalase and Flavourzyme exhibited better antioxidant potential compared to their other-treated and untreated counterparts, indicating the efficacy of employing combinations of endopeptidase and exopeptidase enzymes. Analyses conducted with biological model systems, including the inhibition of hydroxyl and peroxyl radical-induced supercoiled DNA strand scission, cupric ion-induced human LDL peroxidation, and ACE inhibition, further supported the fact that the antioxidative potential of sea cucumber proteins was improved upon enzyme treatment. Therefore, the current study provides a fundamental understanding of using a biorefinery approach for upgrading commercial and underutilized Atlantic sea cucumber tissues, mainly flower, to value-added protein hydrolysates with protective bioactive functions, with a special focus on antioxidant and ACE-inhibitory properties. Nevertheless, further investigations are required in order to characterize the specific peptides responsible for exhibiting antioxidant activity. Additionally, in vivo analysis of protein hydrolysates may further strengthen the findings from in vitro assays.

## Figures and Tables

**Table 1:** Inhibition of hydroxyl and peroxyl radical-induced DNA scission with hydrolysates from the sea cucumber body wall, flower, and internal organs.

Sample	DNA Scission Inhibition (%)	
	Hydroxyl Radical	Peroxyl Radical
BWA	29.61 ± 0.75 ^c^	56.11 ± 1.24 ^c^
BWF	72.18 ± 2.95 ^ab^	69.45 ± 3.90 ^bc^
BWC	69.09 ± 1.18 ^b^	71.19 ± 2.89 ^b^
BW(C+F)	68.61 ± 2.01 ^b^	72.51 ± 2.63 ^b^
BW(A+F)	77.22 ± 2.59 ^a^	81.85 ± 0.18 ^a^
FLA	38.59 ± 2.27 ^d^	57.41 ± 3.53 ^d^
FLF	37.64 ± 2.69 ^d^	63.06 ± 2.47 ^c^
FLC	69.24 ± 0.51 ^b^	72.67 ± 1.32 ^b^
FL(C+F)	72.58 ± 0.65 ^ab^	77.31 ± 0.25 ^ab^
FL(A+F)	76.94 ± 1.09 ^a^	79.79 ± 1.36 ^a^
INA	35.77 ± 1.55 ^c^	57.14 ± 2.64 ^a^
INF	44.48 ± 2.38 ^b^	32.90 ± 1.10 ^c^
INC	58.49 ± 2.50 ^a^	61.53 ± 3.16 ^a^
IN(C+F)	46.71 ± 3.27 ^b^	53. 67 ± 1.47 ^ab^
IN(A+F)	49.49 ± 0.95 ^b^	47.28 ± 2.02 ^b^
Carnosine	77.32 ± 1.42	83.01 ± 0.56

All data represent the mean of triplicate determinations ± standard deviation. Values with different letters in each column are significantly different (*p* > 0.05) for each body part. Abbreviations: BW—body wall; FL—flower; IN—internal organs; A—Alcalase; F—Flavourzyme; C—Corolase; A+F—combination of Alcalase and Flavourzyme; and C+F—combination of Corolase and Flavourzyme. Different letters for the same body part indicate significant differences at *p* < 0.05.

## Data Availability

Not applicable.
